# PCR-Confirmed Convexity Subarachnoid Neurocysticercosis with Negative Serology: A Case Report

**DOI:** 10.3390/pathogens15090996

**Published:** 2026-09-21

**Authors:** Aristos Aristodimou, Zacharias Raptopoulos, Jan Richter, Christina Tryfonos, Anastasia Dimitriadi, Moises Akis Lamprou, Loizos Siakallis

**Affiliations:** 1 Internal Medicine Department, Limassol General Hospital, State Health Organization Services, Limassol 4131, Cyprus; 2 Family Medicine Residency Program, Limassol General Hospital, State Health Organization Services, Limassol 4131, Cyprus; 3 Molecular Virology Department, The Cyprus Institute of Neurology and Genetics, Nicosia 2371, Cyprus; 4 Pathology Department, Nicosia General Hospital, State Health Organization Services, Nicosia 2029, Cyprus; 5 Neurosurgery Department, Nicosia General Hospital, State Health Organization Services, Nicosia 2029, Cyprus; 6 Medical School, University of Cyprus, Nicosia 2029, Cyprus

**Keywords:** neurocysticercosis, *Taenia solium*, extraparenchymal, subarachnoid, seizures, MRI, diagnosis

## Abstract

Extraparenchymal neurocysticercosis, caused by *Taenia solium*, is an uncommon form of neurocysticercosis involving the subarachnoid space and ventricular system. Clinical manifestations are heterogeneous, including seizures, meningitis, and intracranial hypertension, and are often associated with worse outcomes. Diagnosis remains challenging, as cysts may be difficult to detect on conventional imaging due to signal characteristics similar to cerebrospinal fluid, frequently leading to inconclusive results. We describe a patient presenting with a six-month history of focal seizures in whom the diagnosis was established following surgical excision of a cystic lesion, with definitive confirmation provided by positive PCR detection of *T. solium* nuclear ribosomal DNA in the excised tissue. The patient received 14 days of dual antiparasitic therapy and remained free of neurological symptoms during 12 months of follow-up. This case highlights the potential limitations of noninvasive diagnostic modalities and the challenges that physicians might encounter when evaluating infectious diseases that are not endemic to their country of practice. It also underscores the value of a structured diagnostic approach, as recommended by several international health bodies, complemented by advanced molecular techniques such as PCR. Overall, it provides a clinically relevant illustration of key diagnostic challenges and decision-making steps in the evaluation of this complex condition.

## 1. Introduction

Neurocysticercosis (NCC) is caused by the cestode helminth *Taenia solium*, which is also responsible for cysticercosis [1,2]. *T. solium* is currently endemic in sub-Saharan Africa, Latin America, South Asia and Southeast Asia. Although it was historically endemic in several European countries until the early 20th century, endemic transmission has since declined significantly in those countries. Nevertheless, increased migration from endemic regions has led to the detection of cases in European countries (for example, Belgium) [3,4,5]. Human neurocysticercosis results from ingestion of *T. solium* eggs, which are transmitted through the fecal–oral route, whereas ingestion of undercooked pork containing viable cysticerci results in intestinal taeniasis [1,2]. The helminth can form cysticerci in muscles (both skeletal and cardiac), skin and subcutaneous tissues, as well as the central nervous system (CNS) [6].

Infection of the CNS may be either parenchymal (most common) or extraparenchymal (EPNCC), which, according to Fogan and Nash, is further sub-classified into ventricular NCC, subarachnoid NCC of the brain convexity, subarachnoid NCC of the lateral fissure, and basal subarachnoid NCC, owing to differences in pathology, anatomical location, and clinical presentation [7,8]. The presenting symptoms of NCC depend on the location of the cysticerci in the brain, with seizures being the most common, followed by headaches and focal neurological signs [7,9]. Extraparenchymal disease often has a poor prognosis with high rates of mortality and disability [9]. This reflects a robust inflammatory response elicited by cysts within various cerebrospinal fluid (CSF) compartments, causing vasculitis, meningitis, strokes, intracranial hypertension, and death [9].

The diagnostic criteria proposed by Del Brutto et al., widely endorsed by international health bodies such as the Infectious Diseases Society of America (IDSA), integrate histologic, neuroimaging, and clinical/exposure evidence [8]. These criteria allow classification of cases into definitive or probable diagnoses based on the strength and combination of findings (absolute, major, and minor criteria) [10]. The following report describes the case of a young man with EPNCC in whom neuroimaging findings and histopathological examination of an excised brain cyst were suggestive but non-diagnostic, while definitive confirmation of the diagnosis was ultimately achieved by PCR detection of *T. solium* nuclear ribosomal DNA in the excised tissue.

## 2. Case Presentation

A 37-year-old male presented to the Emergency Department (ED) of Limassol General Hospital, Cyprus, complaining of right-sided arm twitching lasting a few minutes. The patient reported multiple similar episodes over the previous six months. He denied confusion, headaches, loss of consciousness, incontinence, slurred speech, dysphagia, diplopia, fever, or recent weight loss. No involvement of other limbs was reported. His past medical history was significant for arterial hypertension, for which he was not receiving regular medication. No known drug allergies were reported. The patient was of Cameroonian origin and immigrated to Cyprus two years earlier, where he works as a private-sector employee. There was no history of smoking, alcohol consumption, or illicit drug use. He denied any recent travel.

Upon initial evaluation, the patient was afebrile and haemodynamically stable. Neurological examination revealed no focal deficits, and the remainder of the physical examination was unremarkable. There was no evidence of urinary or faecal incontinence, tongue biting, or postictal confusion. Preliminary blood tests were significant for leukocytosis (13,440 cells/μL) with neutrophil predominance (11,980 cells/μL), marginally elevated serum creatinine (1.10 mg/dL) with normal urea levels (38 mg/dL), elevated lactate dehydrogenase (LDH) of 334 U/L and C-reactive protein (CRP) of 1.43 mg/L.

**Figure 1:**
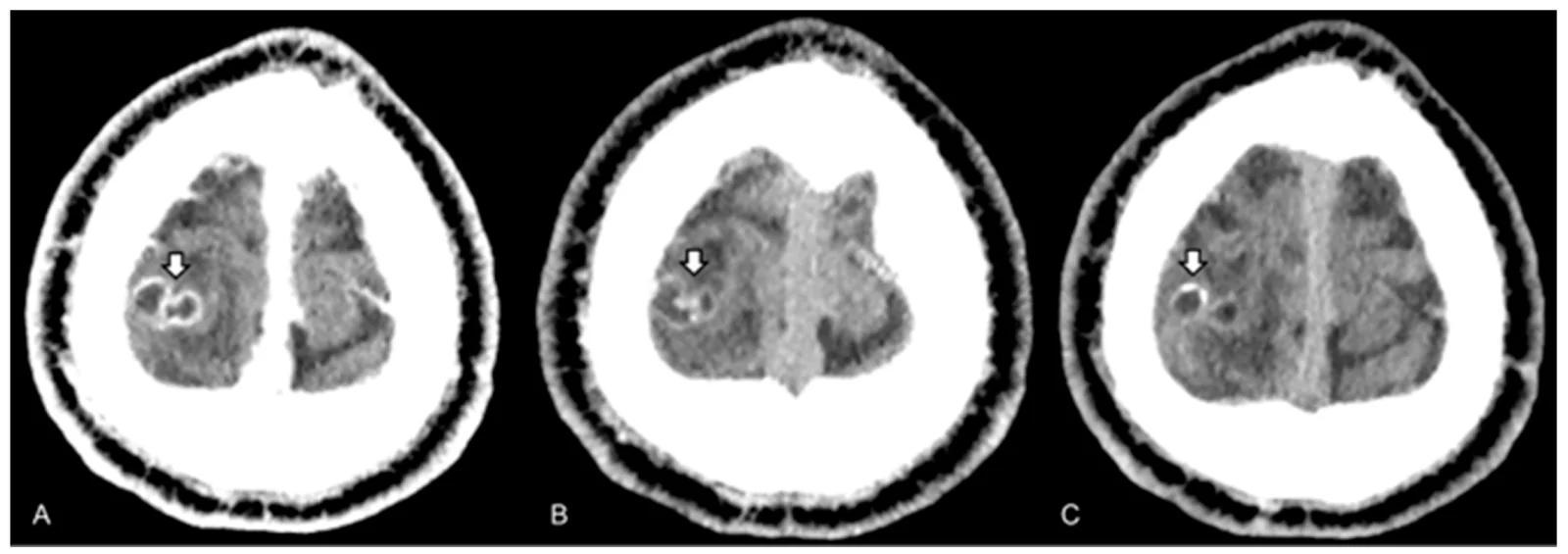
(**A**) Axial contrast-enhanced CT image demonstrates a multilobulated cystic lesion in the right parietal region with peripheral ring-like enhancement (arrow). (**B**,**C**) Axial unenhanced CT images at corresponding (**B**) and slightly higher (**C**) levels demonstrate thin peripheral calcification along the lesion wall (arrows). There is no hyperdensity to indicate gross intralesional haemorrhage. Hypodensity of the surrounding parenchyma indicates vasogenic oedema.

Computed tomography (CT) of the brain revealed a lobulated, ring-enhancing hypodense lesion at the right parietal convexity, measuring approximately 1 × 1.5 cm, with surrounding parenchymal hypodensity consistent with vasogenic oedema (Figure 1). He was commenced on antiepileptic therapy with levetiracetam 500 mg twice daily along with intravenous dexamethasone 0.1 mg/kg/day and was admitted for further investigation and management. Lumbar puncture was not performed because of the marked perilesional cerebral oedema and concern for increased intracranial pressure, with the potential risk of cerebral herniation.

A multichannel, digital electroencephalogram (EEG) recording was performed, with intermittent but well-formed and well-organized alpha rhythm of 10–11 Hz of medium amplitude seen symmetrically over the posterior-temporal regions, reactive to visual attention. Moreover, fast activity of low amplitude was detected over both anterior and central areas, with low-amplitude theta and delta waves bilaterally over the frontocentral regions.

In view of the ring-enhancing hypodense lesion, further investigations were performed to evaluate the main differential diagnoses (Table 1) [11,12]. These included ESR (5 mm/h), serum antibodies for *Toxoplasma gondii* and *Echinococcus granulosus*, serum antigen for *Cryptococcus* spp. and serologic studies for HIV, hepatitis B and syphilis—all of which were negative. A positive hepatitis C antibody test prompted HCV RNA testing that ruled out active infection. To further assess the possibility of sarcoidosis, serum levels of angiotensin-converting enzyme (ACE) were measured and found to be within normal limits (63.6 U/L). Stool examinations were performed for ova, cysts, and parasites to evaluate for concomitant intestinal parasitic infection, particularly *Taenia solium* taeniasis. The examinations were negative, providing no evidence of concomitant intestinal parasitic infection; however, this did not exclude neurocysticercosis.

Bilateral arm and leg radiographs were performed, without evidence of soft tissue calcifications suggestive of cysticercosis.

The initial brain CT raised concerns for metastatic disease; therefore, further imaging of the chest, abdomen and pelvis with CT was requested. In the abdomen, a 4.7 mm hypodense lesion was depicted in the liver (segment VI), along with an intraluminal hypodense lesion in the gastric fundus (4 mm × 1.77 cm) and thickening of the transverse colon. Subsequent gastroscopy and colonoscopy revealed no abnormalities.

**Figure 2:**
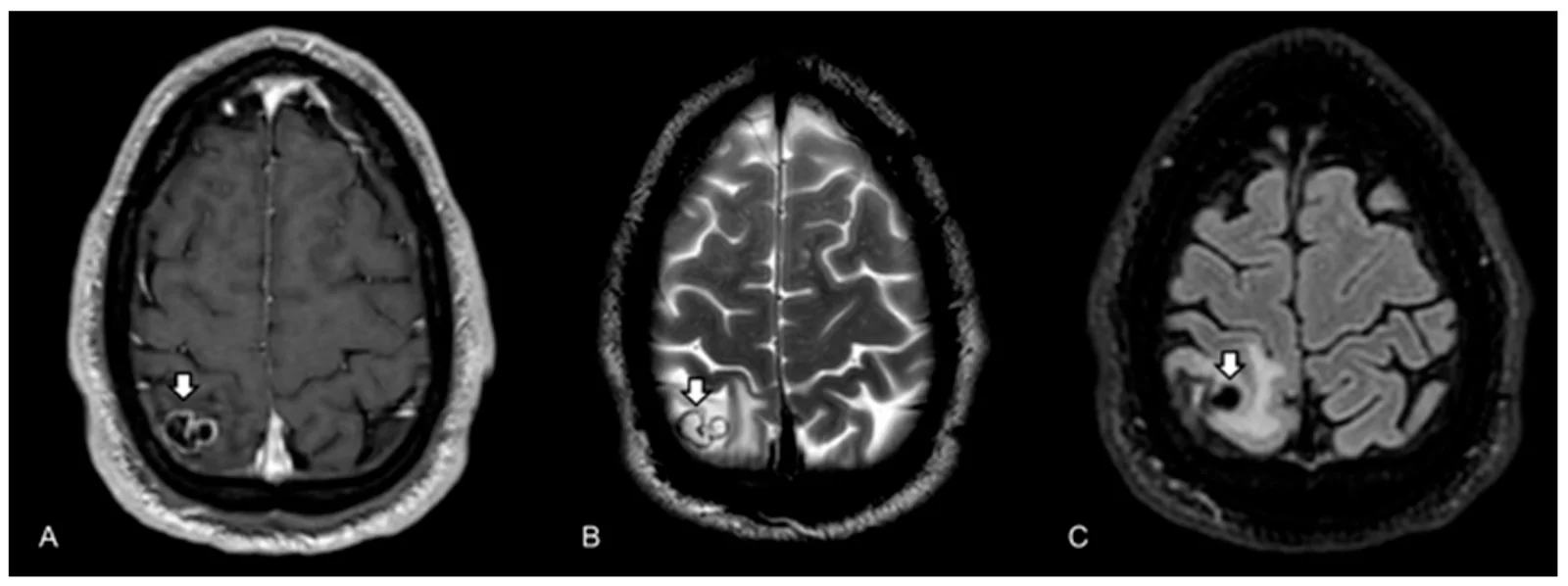
(**A**) Axial post-contrast T1-weighted image demonstrates a lobulated cystic lesion in the right parieto-occipital region with peripheral ring-like enhancement (arrow). (**B**) Axial T2-weighted image demonstrates the cystic lesion and surrounding vasogenic oedema, with decreased signal intensity along the lesion wall (arrow). (**C**) Axial fluid-attenuated inversion recovery (FLAIR) image demonstrates extensive surrounding white matter oedema and decreased signal intensity within the lesion (arrow).

**Figure 3:**
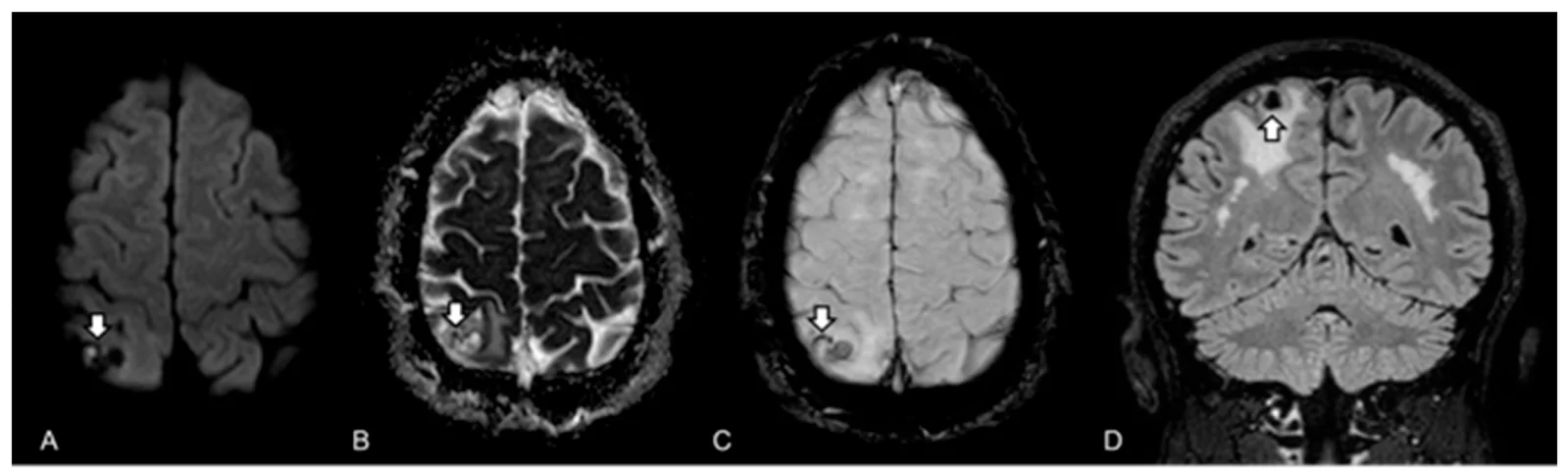
(**A**) Axial diffusion-weighted image (DWI) demonstrates partial restricted diffusion within the lateral component of the lesion (arrow). (**B**) Corresponding apparent diffusion coefficient (ADC) map confirms restricted diffusion within this component, with facilitated diffusion medially. (**C**) Axial susceptibility-weighted image demonstrates peripheral susceptibility predominantly corresponding to the lesion wall (arrow). (**D**) Coronal FLAIR image demonstrates the lesion (arrow) with extensive surrounding white matter oedema.

**Figure 4:**
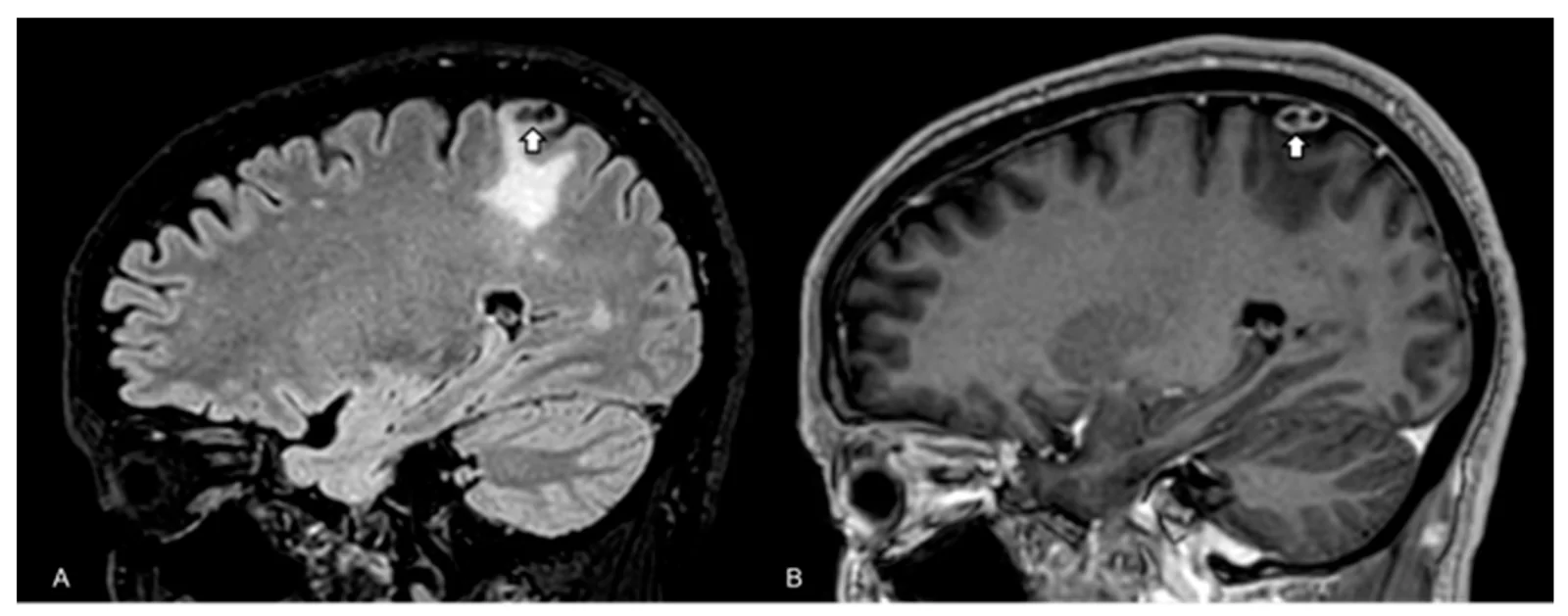
(**A**) Sagittal FLAIR image demonstrates a lobulated cystic lesion over the right parietal convexity (arrow), with extensive vasogenic oedema within the adjacent white matter. (**B**) Sagittal post-contrast T1-weighted image demonstrates peripheral ring-like enhancement of the lesion (arrow) and its apparent extra-axial location, with mild displacement of the adjacent parenchyma.

A magnetic resonance imaging (MRI) of the brain was performed to better visualize and characterize the lesions identified on CT and revealed a right-sided parietal ring-enhancing lesion measuring 1.4 × 1.9 × 1 cm, appearing cortically based but with possible extra-axial extension, with marked perilesional oedema (Figure 2, Figure 3 and Figure 4). Additionally, multiple partly confluent T2 hyperintense lesions in the deep white matter of both cerebral hemispheres, without diffusion restriction or enhancement, were reported. The lesion demonstrated several atypical imaging features. Although initially interpreted as cortically based, sagittal multi-planar reconstructions suggested an extra-axial location. Peripheral susceptibility corresponded to the limited mural calcification identified on CT, while partial restricted diffusion was compatible with degeneration of the lesion. Neurocysticercosis became the primary working diagnosis and, according to the recommended diagnostic criteria, serologic testing for *T. solium* was requested. Given that Cyprus is non-endemic to *T. solium*, serological assays such as EITB and ELISA (antibody/antigen) are not routinely available. Consequently, serum antibody testing by ELISA was conducted on a sample sent abroad, which yielded a negative result. The detailed characteristics of the commercial assay were not available in the clinical documentation and could not be retrospectively confirmed.

**Figure 5:**
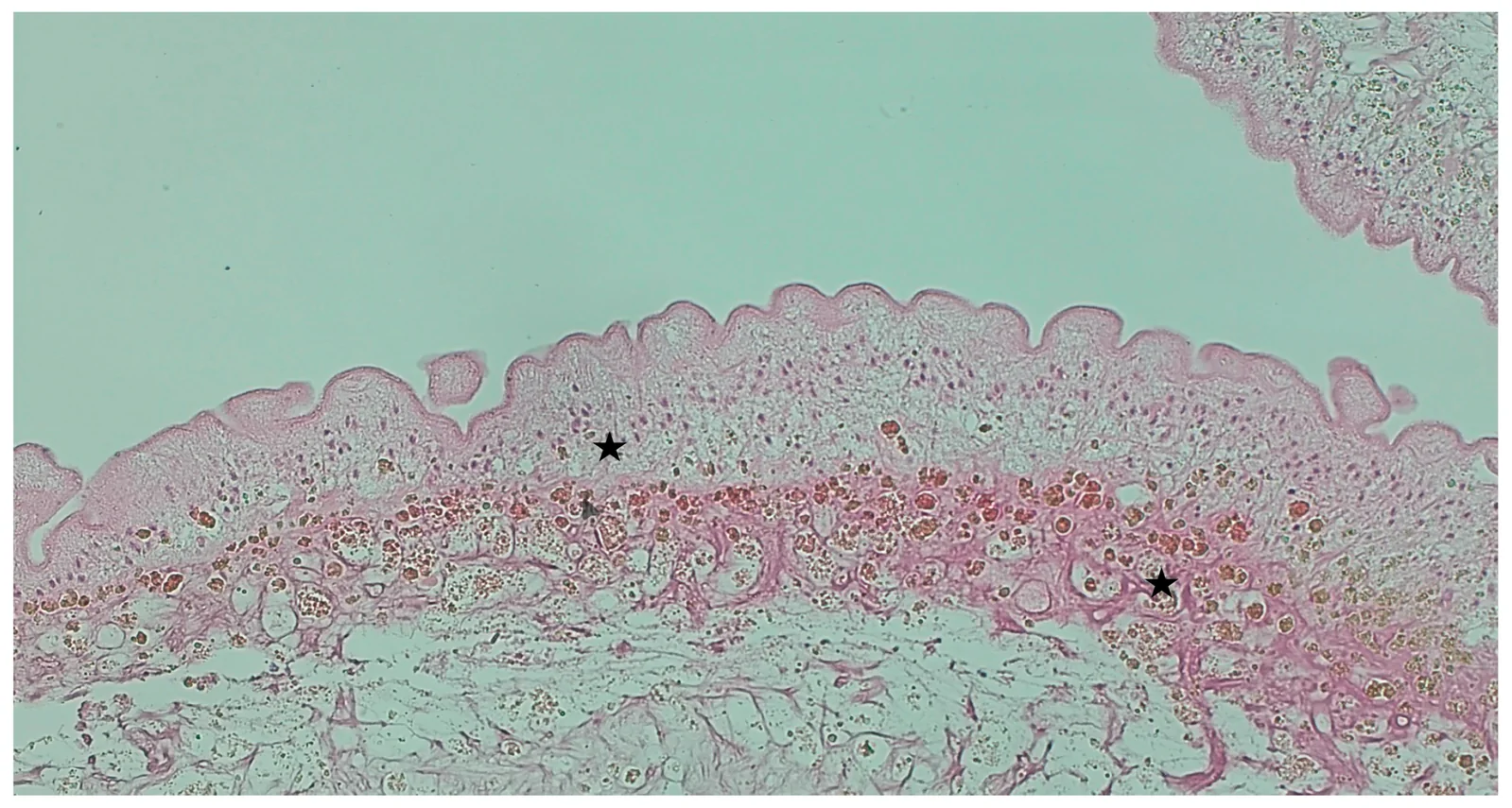
PASX25. Inside the cyst, a structure was seen, with PAS+ capsule (black arrows) and degenerative changes (stars).

**Figure 6:**
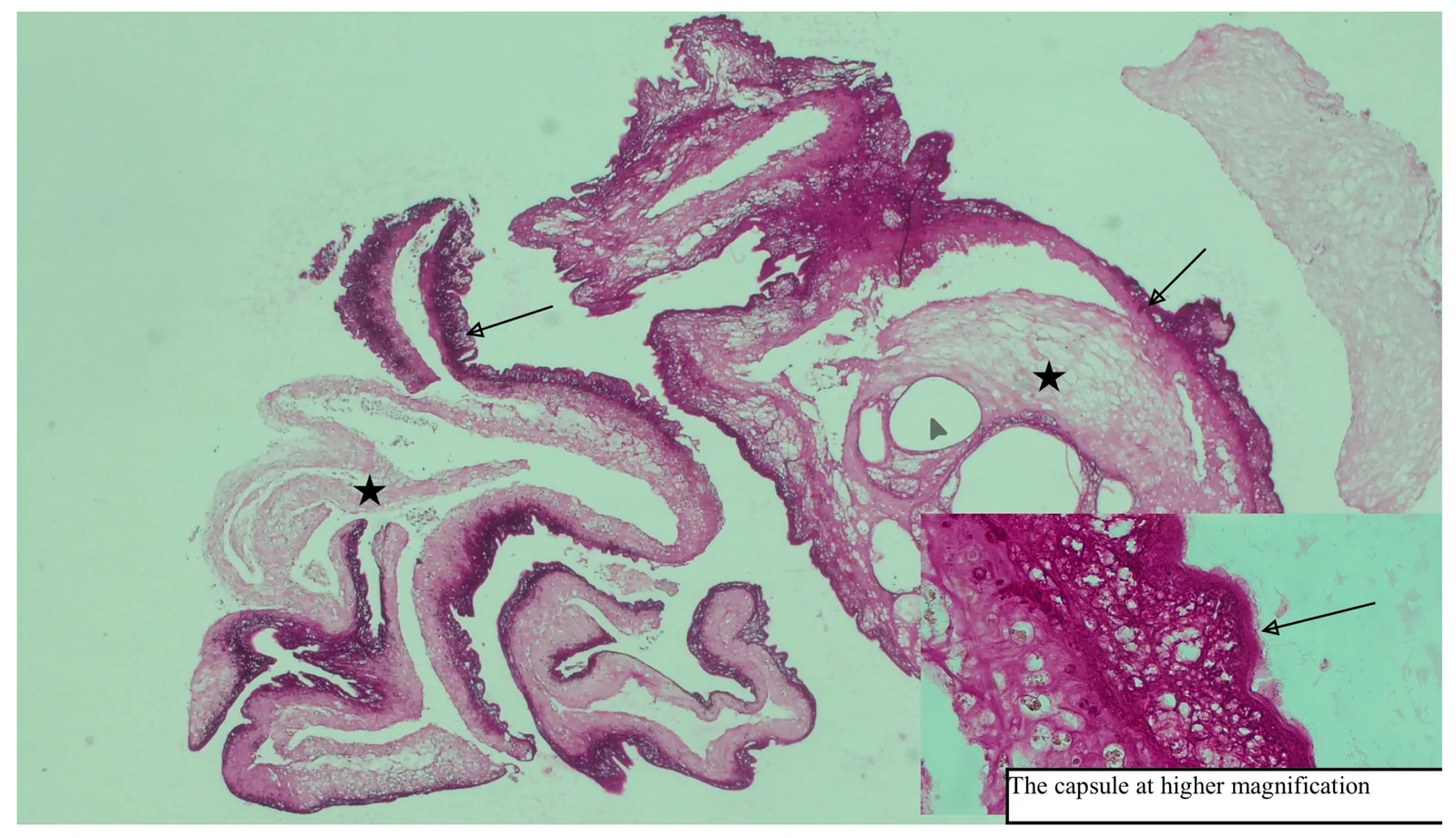
HEX100 Degenerative changes with formation of coarse mineralised granules (stars).

The patient was referred for neurosurgical evaluation and underwent complete surgical excision. The cyst was located in the subarachnoid space. Histopathology analysis showed a cystic lesion measuring approximately 1.1 cm in diameter, which consisted of a fibrous capsule with chronic inflammation consisting of lymphocytes, plasma cells, and histiocytes. Granulomatous reaction was not observed. Inside the cyst, a structure was seen with PAS positive capsule (Figure 5) and degenerative changes, with formation of hyaline granules (Figure 6). The whole structure was reminiscent of a parasite.

The pathology report was given as inflammatory changes, most probably of infectious aetiology and cysticercus was suggested. The pathological findings were suggestive of cysticercosis but were considered non-diagnostic, mainly because of the limited experience with such lesions.

At that point, and prior to initiation of dual antiparasitic therapy, the patient underwent fundoscopy (no ocular involvement) and screening for latent tuberculosis (negative tuberculin skin test), *Strongyloides stercoralis* and *Schistosoma* spp. (all negative).

Following complete surgical excision, the patient received albendazole and praziquantel for 14 days. This treatment was probably not strictly necessary, but since it was not possible to completely rule out the presence of a residual or additional lesion, it was decided to administer it empirically. Dexamethasone was initiated before antiparasitic therapy because of the strong clinical and radiological suspicion of neurocysticercosis and the presence of substantial perilesional oedema, followed by rapid tapering. Following surgical excision, the lesion was ultimately classified as EPNCC-SAS based on its subarachnoid location, histopathological assessment, and molecular confirmation.

Furthermore, the surgically excised tissue was subsequently submitted for molecular analysis. DNA was extracted from formalin-fixed, paraffin-embedded tissue using the MagPurix FFPE DNA Extraction Kit (Zinexts Life Science Corp., Taiwan, China), according to the manufacturer’s instructions. *T. solium* DNA was detected using the *T. solium*-specific component of the TaqMan real-time PCR described by Ng-Nguyen et al. [13]. The assay targets the ITS-1 region of nuclear ribosomal DNA, a non-coding spacer located between the 18S and 5.8S rRNA-encoding regions. The primers were 5′-TGTTAGCAGCAGTTGTGATG-3′ and 5′-CCAACTCCACCCAGATTGA-3′, with the species-specific probe FAM-5′-CCAGCGCTGCTGTTGACTGA-3′-BHQ1. The expected amplicon was approximately 92 bp. Each run included a *T. solium* positive control and a no-template negative control. A result was considered positive when species-specific exponential amplification occurred at Ct ≤ 40 and all controls fulfilled the predefined acceptance criteria.

Following PCR confirmation, retrospective re-evaluation of the histopathological sections, based on the observed degenerative changes and hyaline granules, was considered compatible with the granular–nodular stage of neurocysticercosis, although the parasite itself was not directly demonstrated.

The patient was followed clinically for 12 months after treatment and remained symptom-free, with no further seizures or new neurological symptoms. Levetiracetam was continued for 12 months, during which no recurrent seizures were reported. A follow-up brain MRI was recommended and the patient was referred for repeat imaging; however, this was not subsequently performed because the patient did not attend the scheduled appointment. Consequently, although the subarachnoid lesion had been completely excised surgically, the absence of follow-up MRI precluded assessment of possible radiological recurrence or the development of new cystic lesions. No treatment-related adverse effects were documented during follow-up, and serial full blood counts and liver function tests remained within reference ranges.

## 3. Discussion

Neurocysticercosis (NCC) is the most common parasitic disease of the central nervous system (CNS), with an estimated 1.37 million disability-adjusted life years (DALYs) worldwide [14]. Cysticercosis should not be regarded as a tropical disease per se. Although it is currently most prevalent in low- and middle-income regions of Latin America, sub-Saharan Africa, and South and Southeast Asia, cysticercosis was endemic in many European countries during the nineteenth and early twentieth centuries [3,4]. Its distribution is closely linked to socioeconomic conditions and sanitation, as reflected in the life cycle of *Taenia solium*, in which human fecal contamination and inadequate sanitation facilitate transmission. *T. solium* has also been identified in European countries in recent years, including Cyprus, where this represents the second imported case within a two-year period, highlighting the impact of population mobility on the redistribution of infectious diseases [15]. Extraparenchymal NCC (EPNCC) is uncommon and may have diverse presentations with more subtle imaging findings, most likely because the cystic fluid has signal intensity similar to CSF, thereby impeding identification of racemose cysts where scolices are not present [16].

In 2016, diagnostic criteria specifically distinguishing parenchymal from extraparenchymal neurocysticercosis were published and validated, representing the first diagnostic criteria to formally differentiate these two major forms of the disease [17]. This distinction is clinically relevant because extraparenchymal neurocysticercosis differs from parenchymal disease in its anatomical distribution, imaging characteristics, clinical manifestations, and therapeutic considerations. The diagnosis of NCC is also guided by the revised criteria proposed by Del Brutto and colleagues in 2017, which consider various clinical variables such as neuroimaging findings, clinical/epidemiological evidence, the presence of antibodies/antigens in the CSF or in the blood, and the identification of the Cysticercus upon histopathologic evaluation [1,2]. With regard to serological tests, two techniques are primarily used: ELISA and EITB. Although most studies have reported that the EITB is more effective than ELISA at detecting specific antibodies [2,18,19], other studies have found no significant difference between the two techniques [20,21]. EITB was not available in our setting, so serum *T. solium*-specific IgG antibodies were therefore assessed using the available ELISA assay. The negative result was interpreted in the context of the limited sensitivity of antibody-based testing and did not exclude NCC. Initial neuroimaging findings demonstrated a lobulated hypodense lesion on CT with peripheral ring enhancement, limited mural calcification and hypodensity of the surrounding parenchyma, indicating vasogenic oedema. MRI confirmed a ring-enhancing lesion centred at the right parietal convexity with marked surrounding oedema. This was initially considered cortically based; however, on review, it appeared likely extra-axial on multi-planar reconstructions. An important clinicoradiological consideration in this case is the apparent discrepancy between the patient’s seizure semiology and the anatomical location of the lesion. The patient experienced isolated paroxysmal twitching of the right upper limb, whereas the identified lesion was located in the right parietal region, which does not provide a straightforward anatomical correlate given the predominantly contralateral organization of the motor pathways [22]. The bilateral deep white-matter abnormalities were also unlikely to represent a specific seizure focus, given their diffuse distribution, deep location, and absence of enhancement or diffusion restriction. Although white-matter abnormalities may potentially contribute to altered cerebral network excitability in epilepsy, their contribution to the patient’s clinical presentation cannot be established from the available data. Similarly, the cortical/possible extra-axial location of the right parietal lesion and its marked perilesional oedema provide a plausible substrate for cortical irritation and seizure generation, consistent with the recognised association between perilesional oedema and seizure activity in neurocysticercosis [23,24]. However, a direct clinicoradiological correlation cannot be confirmed in this case. An EEG demonstrated a preserved, symmetric posterior alpha rhythm with nonspecific low-amplitude bilateral frontocentral theta and delta activity, but no reported epileptiform discharges or electrographic seizures. Thus, electrophysiological confirmation or localization of the reported episodes was not established. Although these findings raised suspicion for NCC, they were not considered sufficient to establish a definitive diagnosis according to the revised Del Brutto criteria. Thus, the patient was referred for surgical excision, which revealed that the lesion was actually located in the subarachnoid space. Histopathologic analysis of the tissue was significant for inflammatory changes, most likely involving the meningeal layer, suggesting an underlying infectious aetiology, with NCC as the most likely diagnosis, but without absolute demonstration of the parasite as suggested by Del Brutto et al. (presence of cysticerci in biopsy material, including the spiral canal and the rostellum with its four suckers and the double crown of hooks, or the typical three-layered membrane wall that is a characteristic pathological finding of EPNCC) [10]. The intraoperative identification of the cyst within the subarachnoid space provided an important anatomical clue and allowed the lesion to be retrospectively interpreted as a multilobulated subarachnoid cystic lesion. However, we do not consider this finding to represent a second independent major neuroimaging criterion in addition to the ring-enhancing appearance, as both findings describe morphological and anatomical characteristics of the same lesion. Moreover, the subarachnoid location was established intraoperatively rather than unequivocally demonstrated on the initial neuroimaging studies. Accordingly, the Del Brutto criteria were useful in supporting the clinical suspicion of NCC but were not considered sufficient to establish a definitive diagnosis in this case [10]. Importantly, the diagnosis was ultimately confirmed by molecular analysis of the excised tissue. TaqMan real-time PCR targeting the ITS-1 region of nuclear ribosomal DNA provided definitive molecular confirmation of the aetiological diagnosis. Thus, the surgical and histopathological findings should be regarded as supportive and complementary evidence, whereas PCR provided the definitive confirmation of *T. solium* infection.

Diagnosis of EPNCC is often challenging because lesions may not be clearly identified on MRI [21]. Parasites in these locations typically exhibit signal characteristics similar to CSF, usually do not enhance with intravenous contrast administration, and commonly lack a visible scolex [25]. Consequently, extraparenchymal NCC may be overlooked on conventional MRI or manifest only through indirect findings such as arachnoiditis or distortion of the subarachnoid spaces. Dedicated three-dimensional heavily T2-weighted sequences, including FIESTA or CISS, can improve detection of subarachnoid cysticerci [25,26,27]. In the present case, multi-planar MRI reconstructions suggested an extra-axial location that was not initially evident on axial imaging alone. On retrospective review, the combination of a lobulated extra-axial lesion, peripheral ring enhancement, limited mural calcification on CT with corresponding susceptibility on MRI, partial restricted diffusion, and extensive surrounding vasogenic oedema was compatible with degeneration of an extraparenchymal cyst. When considered together with the histopathological evidence of hyaline degeneration and coarse mineralised granules, the findings were compatible with a cysticercus in the granular–nodular stage. These imaging characteristics further complicate the diagnosis of EPNCC, particularly in non-endemic regions where clinicians and radiologists may be less familiar with the disease’s often subtle and atypical presentations [26]. Surgical excision, prompted by the diagnostic uncertainty, provided anatomical evidence supporting extraparenchymal NCC, while histopathology demonstrated nonspecific inflammatory and degenerative changes and *T. solium*-specific TaqMan real-time PCR targeting the ITS-1 region of nuclear ribosomal DNA provided definitive molecular confirmation of the diagnosis. Molecular detection of *T. solium* has been described as a useful diagnostic approach in challenging cases in which conventional diagnostic methods are inconclusive (Table 2) [25,28,29,30,31].

This case also has implications for the diagnostic approach to suspected neurocysticercosis in non-endemic settings. The Del Brutto criteria have provided an important framework for standardizing the diagnosis of neurocysticercosis, but their application may be challenging in atypical extraparenchymal disease, in which characteristic imaging features such as a visible scolex may be absent and serological testing may be negative [2,10]. Proposed revisions have sought to make the diagnostic framework more operational and to place greater emphasis on characteristic neuroimaging findings, although their validity remains incompletely established [18].

Although surgery should not be routinely pursued solely because neurocysticercosis is suspected or EITB is unavailable, tissue diagnosis may become appropriate when a surgically accessible lesion remains indeterminate after comprehensive non-invasive evaluation and important alternative diagnoses requiring different management cannot be adequately excluded [2]. In such circumstances, molecular testing of excised tissue may provide definitive confirmation when conventional histopathology is non-diagnostic. Thus, in non-endemic settings, a multidisciplinary diagnostic approach involving neuroradiology, infectious diseases, neurology, neurosurgery, pathology, and, where available, molecular diagnostics may be particularly valuable.

Several limitations should be acknowledged. First, although follow-up brain MRI was recommended and the patient was referred for repeat imaging, it was not subsequently performed; therefore, radiological resolution or persistence of the lesion could not be assessed. Second, lumbar puncture was not performed because of the marked perilesional cerebral oedema and concern for increased intracranial pressure, with the potential risk of cerebral herniation. Consequently, cerebrospinal fluid analysis was unavailable to further evaluate potential infectious or inflammatory alternative diagnoses. Nevertheless, the diagnosis was ultimately established through histopathological examination and definitive molecular confirmation by PCR detection of *T. solium* nuclear ribosomal DNA in the excised lesion.

**Figure 7:**
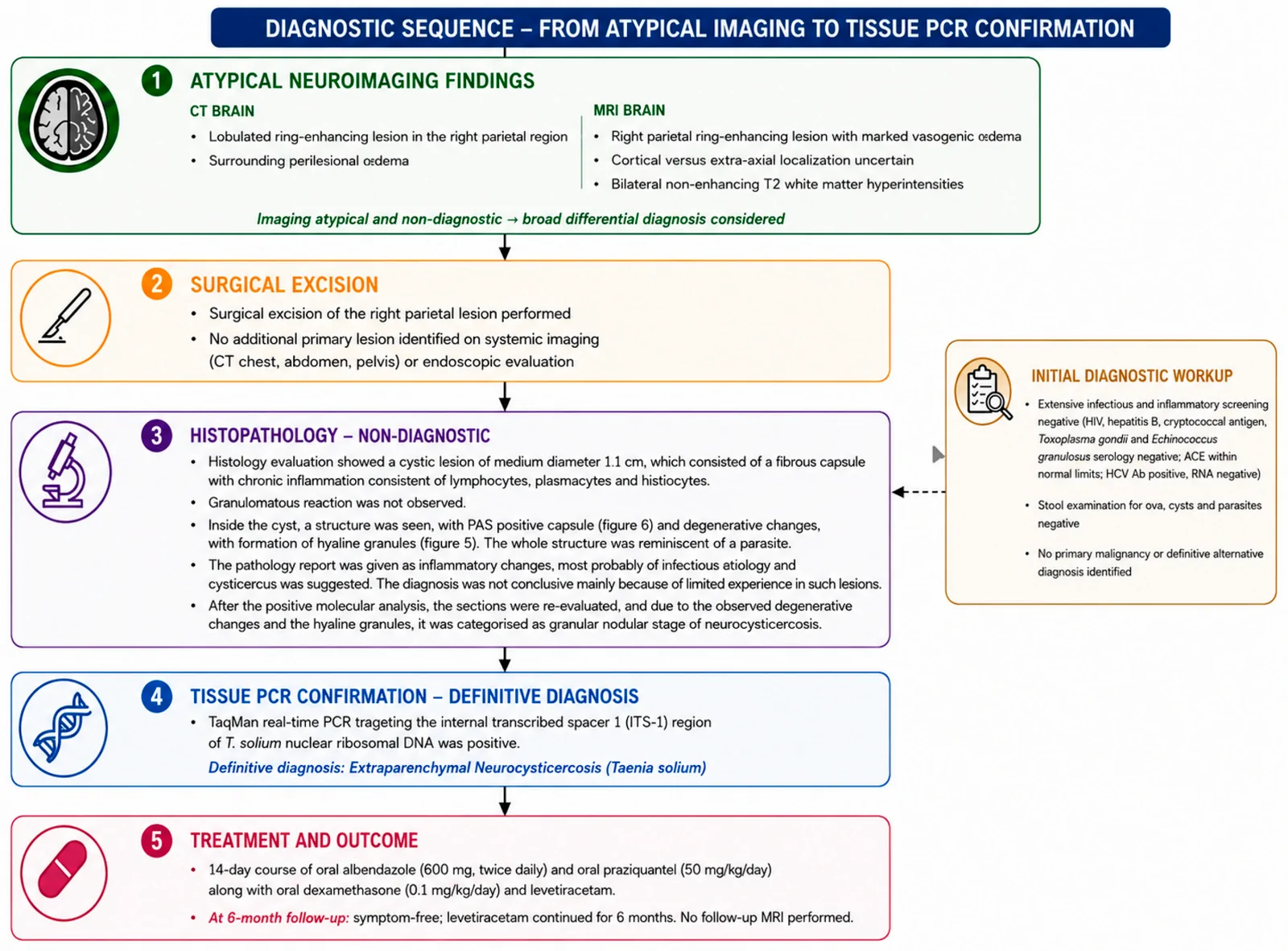
Diagnostic sequence.

This case illustrates how diagnostic uncertainty—driven by nuanced neuroimaging findings and negative serological studies—can ultimately lead to surgical intervention to characterize the lesion and obtain diagnostic tissue (Figure 7). The anatomical information obtained during surgery and the histopathological findings provided important supportive evidence, but definitive aetiological confirmation was achieved by PCR detection of *T. solium* nuclear ribosomal DNA in the excised tissue (Table 3). This distinction is particularly relevant in atypical extraparenchymal NCC, where conventional neuroimaging and serological investigations may not provide definitive evidence of infection.

***Diagnostic schema***

## 4. Conclusions

Extraparenchymal neurocysticercosis poses a substantial diagnostic challenge, particularly in non-endemic countries where clinical suspicion may be low and established diagnostic criteria may not be readily fulfilled. This case demonstrates the limitations of conventional neuroimaging and serological investigations in detecting and characterizing subarachnoid disease, where cysts may exhibit signal characteristics similar to cerebrospinal fluid and lack characteristic features such as a visible scolex. In non-endemic settings, a negative serum serological assay does not exclude extraparenchymal neurocysticercosis when the clinical and radiological context remains compatible. Definitive diagnosis in this case was achieved only after surgical excision, with molecular confirmation of *Taenia solium* DNA in the excised tissue, thereby resolving the diagnostic uncertainty created by the atypical imaging and non-diagnostic histopathology. In carefully selected patients with a surgically accessible lesion that remains indeterminate despite appropriate non-invasive evaluation, particularly when important alternative diagnoses cannot be excluded, surgical tissue sampling or excision with subsequent molecular testing may provide definitive diagnosis and, where appropriate, therapeutic management. Recognition of these diagnostic limitations is essential to avoid delayed diagnosis and inappropriate management.

## Figures and Tables

**Table 1:** Differential diagnosis for a ring-enhancing lesion.

Diagnosis Considered	Evidence for	Evidence Against
**Neurocysticercosis**	Cystic/ring-enhancing morphology; mural calcification; marked perilesional oedema; degenerative features; eventual PCR confirmation	Atypical convexity/possible extra-axial location; absence of a typical scolex
**Primary CNS tumour**	Ring enhancement; possible extra-axial/convexity appearance; surrounding oedema	Cystic morphology with mural calcification and degenerative features; definitive pathology did not support neoplasia
**Metastasis**	Ring enhancement and perilesional oedema	Solitary lesion; no known primary malignancy; atypical morphology; definitive pathology negative
**Bacterial abscess**	Ring enhancement, oedema and diffusion abnormality	No clinical evidence of systemic infection; only partial rather than marked diffusion restriction; atypical morphology
**Tuberculoma**	Ring enhancement and possible calcification	No supportive clinical/systemic findings; morphology and location atypical; definitive pathology negative
**Toxoplasmosis**	Ring-enhancing CNS lesions can occur with surrounding oedema	Solitary lesion and clinical context not suggestive; no evidence of immunocompromised state
**Fungal infection**	Can produce ring-enhancing lesions	No relevant clinical risk factors; morphology and clinical presentation atypical
**Demyelinating disease**	Inflammatory/demyelinating lesions may demonstrate ring enhancement	Cystic morphology, mural calcification and marked oedema were atypical; pathology negative
**Other parasitic infection**	Several parasitic infections can produce cystic or ring-enhancing lesions	Imaging and definitive molecular findings supported *T. solium*

**Table 2:** Published cases of neurocysticercosis with atypical diagnostic findings and/or molecular confirmation.

*Author, Year*	*Patient*	*Lesion Location*	*Imaging Findings*	*Serology*	*Histopathology*	*Molecular Method*
***Ong et al. [29], 2020***	35-year-old male presented with first-onset generalised seizure	Left temporal lobe lesion	MRI of the brain revealed a solitary ring-enhancing lesion measuring 1.2 by 1 cm	Positive	Granulomatous inflammation. Aggregates of epithelioid histiocytes and multinucleated giant cells lining the fibrous cyst wall, accompanied by a mixed inflammatory infiltrate and necrosis. Features characteristic of a cysticercus were absent, and no viable larva was identified	pan-Cestoda PCR targeting a fragment of the mitochondrial ribosomal RNA genes showed 99.6% similarity, thus confirming the presence of *T. solium* DNA
***Chotmongkol et al. [30], 2018***	41-year-old male with progressive vision and hearing loss on the right side for 3 years.	Multiple lesions at the quadrigeminal, prepontin cisterns and lateral ventricles.	MRI of the brain showed multiple small cystic lesions at the quadrigeminal, prepontine cisterns, and lateral ventricles with heterogeneous enhancement, and ring-enhancing lesions in the right optic and internal acoustic canals	Non-reactive ELISA	Not obtained	Not performed
***Lordick et al. [31], 2026***	43-year-old woman with a first-time generalised epileptic seizure	Bihemispheric ring-enhancing lesions	MRI showed a bihemispheric pattern of eight small (3–8 mm), ring-enhancing lesions with surrounding white matter oedema without mass effect	EITB was negative	Brain biopsy revealed no evidence of malignancy, but showed granulomatous inflammation with eosinophilic infiltrates, foreign-body giant cells, and amorphous material suggestive of parasitic remnants	*T. solium* DNA was detected using pTsol9-specific DNA-based PCR
***Fan et al. [25], 2018***	53-year-old woman with recurrent headache, blurred vision and progressive memory loss	Extraparenchymal and parenchymal disease	Brain MRI showed hydrocephalus and diffuse T2-weighted hyperintensity in the juxta-ventricular white matter, together with enhancement of the meninges, and multiple cystic lesions in the prepontine cistern, ambient cistern, and suprasellar cistern	Positive serum and CSF assay for IgG	Not performed	Not performed
***Fan et al. [25], 2018***	57-year-old man with paroxysmal blurred vision for 2 months	Parenchymal and possibly extraparenchymal NCC	Head CT revealed a single calcified lesion in the left frontal lobe	Positive serum and CSF assay for IgG	Not performed	PCR of CSF identified *T. solium* DNA.

**Table 3:** Take-Home Messages.

Take-Home Messages
Extraparenchymal neurocysticercosis can present with atypical neuroimaging features, making diagnosis challenging. Lesions in subarachnoid spaces may be difficult to characterize, may resemble other cystic or ring-enhancing lesions, and may lack a visible scolex.
Molecular testing can provide definitive diagnostic confirmation when neuroimaging and histopathology are inconclusive. In this case, TaqMan real-time PCR targeting the ITS-1 region of *Taenia solium* nuclear ribosomal DNA established the diagnosis following surgical excision.
Ring-enhancing CNS lesions have a broad differential diagnosis, encompassing infectious, neoplastic, inflammatory, and parasitic aetiologies; their evaluation requires systematic clinical and radiological assessment and, when appropriate, multidisciplinary collaboration.
Neurocysticercosis should remain in the differential diagnosis in patients from endemic regions, even when serological testing is negative, particularly when imaging findings are atypical or non-specific.

## Data Availability

No new data were created or analyzed in this study.

## Abbreviations

The following abbreviations are used in this manuscript:

NCC	Neurocysticercosis
CNS	Central nervous system
EPNCC	Extraparenchymal neurocysticercosis
CSF	Cerebrospinal fluid
IDSA	Infectious Diseases Society of America
ED	Emergency department
CRP	C-reactive protein
CT	Computed tomography
ACE	Angiotensin-converting enzyme
MRI	Magnetic resonance imaging
DALYs	Disability adjusted life years
EITB	Enzyme-linked Immunoelectrotransfer blot
